# Divergent *Megrivirus* genome detected from wild birds in Brazil

**DOI:** 10.1128/mra.00549-25

**Published:** 2026-08-31

**Authors:** James Lima Ferreira, Edivaldo Costa Sousa Junior, Jessylene Almeida Ferreira, Poliana da Silva Lemos, Fernando Neto Tavares, Mirleide Cordeiro dos Santos, Marcio Roberto Teixeira Nunes, Rita Catarina Medeiros Sousa

**Affiliations:** 1 Section of Virology, Evandro Chagas Institute, Ministry of Health 89124 , Ananindeua, Brazil; 2 Faculty of Public Health at the University of São Paulo 54546, São Paulo, Brazil; 3 Center of Technological Innovation, Evandro Chagas Institute, Ministry of Health 89124 , Ananindeua, Brazil; 4 Nucleus of Tropical Medicine, Federal University of Pará 37871, Belém, Brazil; Katholieke Universiteit Leuven, Leuven, Belgium

**Keywords:** *Megrivirus*, *Picornaviridae*, genome, wild birds, poultry, sequencing, polyprotein, surveillance, pathogens, Brazil

## Abstract

We report the near-complete genome of a divergent *Megrivirus* strain (avian/JF05/BA/BRA) detected in cloacal swabs collected from wild birds in Northeast Brazil. Genome assembly and annotation revealed a 9,680 bp RNA genome with moderate similarities up to 61.8% on the nucleotide level with members of the species *Megrivirus aturhepa*. The study highlights the importance of viral surveillance in wild birds.

## ANNOUNCEMENT

The *Picornaviridae* family is composed of 158 species grouped in 68 genera (1). One of them, the genus *Megrivirus*, is divided into five species: *aturhepa*, *berockdo*, *chigalli*, *ceharri*, and *ipengu*. The genome is a linear, single-stranded, positive-sense RNA molecule of about 9 kb that encodes a polyprotein (2–4). *Megrivirus* is frequently identified in poultry and wild birds, constituting a potential threat when spreading to other animals and humans (5, 6).

In this study, we report the near-complete genome of the divergent *Megrivirus aturhepa* JF05 strain avian/JF05/BA/BRA obtained from a pool of cloacal swabs collected from 10 wild birds of the species *Sterna dougallii*, collected from July and September 2016 to March and April 2017 in Jandaíra, Bahia, Northeast Brazil.

**Fig 1:**
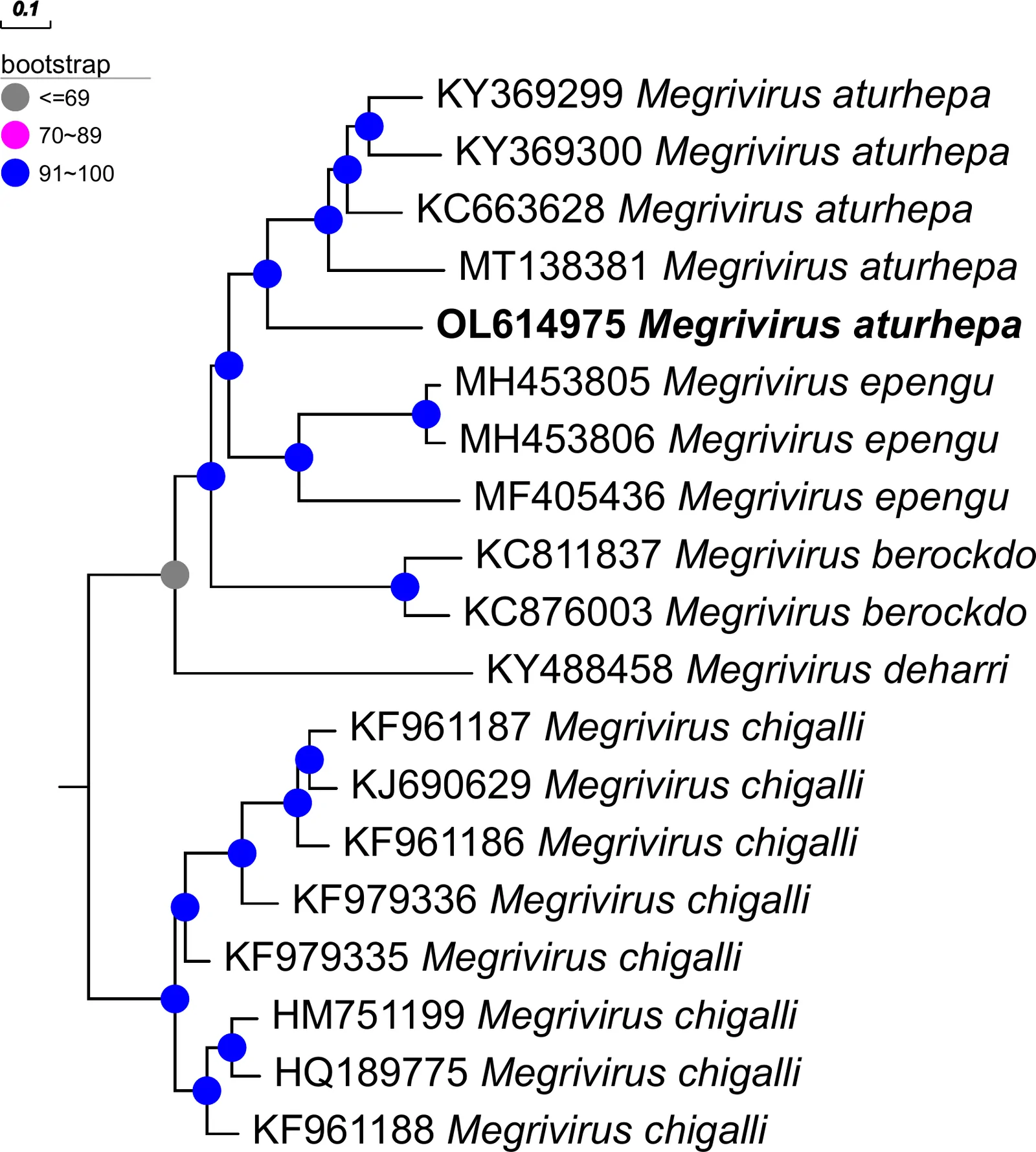
Maximum likelihood (ML) phylogenetic tree of the *Megrivirus aturhepa* JF05 strain obtained in this study (OL614975, highlighted in bold). Only complete and near-complete reference genomes of different *Megrivirus* species retrieved from ICTV and GenBank were aligned using MAFFT v7. The ML tree was reconstructed with FastTree v2.1.11 under the GTR + G model. Node support was assessed with 1,000 bootstrap replicates, and midpoint rooting was applied for visualization. Bootstrap values are indicated by colors. Tree visualization was performed using the EvolView webserver. All parameters were run in default mode unless otherwise specified.

The nucleic acid was extracted from a pool of samples using the QIAamp DNA Mini Kit (QIAGEN). The acid nucleic concentration, used for sequencing, was constructed using 1 ng/μL (Qubit fluorometer, Thermo Fisher Scientific). A paired-end library (2 × 101) was constructed using the Nextera XT DNA Library Prep Kit (Illumina), and sequencing was performed on the HiSeq 2500 sequencing systems (Illumina). The DNA sequencing generated 11,607,371 reads, which were trimmed using the Trimmomatic software v0.39 (7) and assembled by a *de novo* strategy using Megahit software v1.2.9 (8), producing 29,790 contigs ranging from 201 to 385,747 bp. These contigs were taxonomically annotated with Kraken2 v2.1.0 (9), and one contig of 9,680 bp was classified as a member of *Megrivirus aturhepa* A2, presenting 61.8% nucleotide identity to the reference strain LY (KC663628). The genome identified had a coverage of about 115.0×, forming the near-complete genome sequence. The genome alignment and curation were performed with Geneious Prime v2025.2.1 (10). The genome annotation and manual curation were performed using 18 *Megrivirus* genome references retrieved from ICTV and GenBank (KY369299/KY369300/KC663628/MT138381/MH453806/MH453805/MF405436/KC876003/KC811837/KY488458/HM751199/HQ189775/KF979336/KF979335/KF961188/KJ690629/KF961186/KF961187). The resulting genome of *Megrivirus aturhepa* strain avian/JF05/BA/BRA possesses a linear genome 9,594 bp in length with a 47.0% GC content, encoding a single polyprotein. The same 18 genomes were also used for the phylogenetic analysis performed with FastTree software v2.1.11 (11). All bioinformatic tools were run with default parameters unless otherwise specified. Compared with all *Megrivirus* reference genomes from ICTV and GenBank, the polyprotein regions P1, P2, and P3 showed the highest nucleotide identities of 63.7%, 61.6%, and 66.1%, respectively (all values corresponding to the *Megrivirus aturhepa* species). The phylogenetic analysis shows that all species form well-supported groups, with bootstrap values above 91%. Strain JF05 forms an outgroup of the *Megrivirus aturhepa* species (Fig. 1). The results suggest that the JF05 strain could be a divergent strain of *Megrivirus aturhepa* or may represent a new species and demonstrate the importance of monitoring picornaviruses in poultry and wild birds in order to discover new pathogens that can cause problems in agriculture and lead to implications for human health.

## Data Availability

The genome sequence was deposited in GenBank under accession number OL614975, and the raw sequencing reads are available under accession number SRR16964286.
